# Malignant Peripheral Nerve Sheath Tumor Presenting as Horner′s Syndrome

**DOI:** 10.7759/cureus.18341

**Published:** 2021-09-28

**Authors:** Mohamed Azharudeen, Jayachandran Selvaraj, Vivekanandan Pillai, Jeyakumar Meyyappan, Vamsidhar Veeranki

**Affiliations:** 1 General Medicine, Jawaharlal Institute of Postgraduate Medical Education and Research, Pondicherry, IND

**Keywords:** neurofibromatosis 1, malignant peripheral nerve sheath tumour, horner’s syndrome, brachial plexopathy, sarcoma soft tissue

## Abstract

Malignant peripheral nerve sheath tumor (MPNST) is a rare, aggressive sarcomatous tumor that arises from peripheral nerve sheath and shows Schwann cell differentiation. They are commonly seen among cases with existing benign plexiform neurofibromas, prior radiation treatment, and large germline mutations involving the entire neurofibromatosis 1 (NF1) gene. MPNST can have varied presentations; hence diagnosis remains a great challenge. Here we report a rare case of MPNST in an NF1 patient who presented with Horner´s syndrome. A young male reported swelling in the neck, dyspnea on exertion, and dysphagia. Subsequently, he was diagnosed to have a malignant peripheral nerve sheath tumor arising from the mediastinum and compressing the ipsilateral cervical sympathetic plexus causing Horner′s syndrome. The patient underwent surgical resection of the mediastinal mass followed by chemotherapy. His symptoms improved significantly following treatment. This case report emphasizes the fact that high suspicion of MPNST is required when NF1 cases present with mass lesions, so that early surgical clearance with chemoradiation may offer a near-complete cure.

## Introduction

Malignant peripheral nerve sheath tumor (MPNST) is a very rare entity. Neurofibromatosis 1 (NF1) is characterized by various cutaneous, neoplastic, skeletal, and neurological manifestations. The MPNST accounts for approximately 5%-10% of all soft tissue sarcomas, about 50% occur with NF1 [1]. The most common anatomic sites include the proximal portions of the upper and lower extremities and the trunk [1]. We present here a case of NF1 where a malignant peripheral nerve sheath tumor presented as neck swelling and Horner′s syndrome.

## Case presentation

A 30-year-old man presented with swelling on the left side of the neck for six months. He had a history of progressive dysphagia to solids for four months, and dyspnea on exertion for one month. He also had decreased appetite and weight loss for four months. His mother and two siblings were also diagnosed to have NF-1.

**Figure 1:**
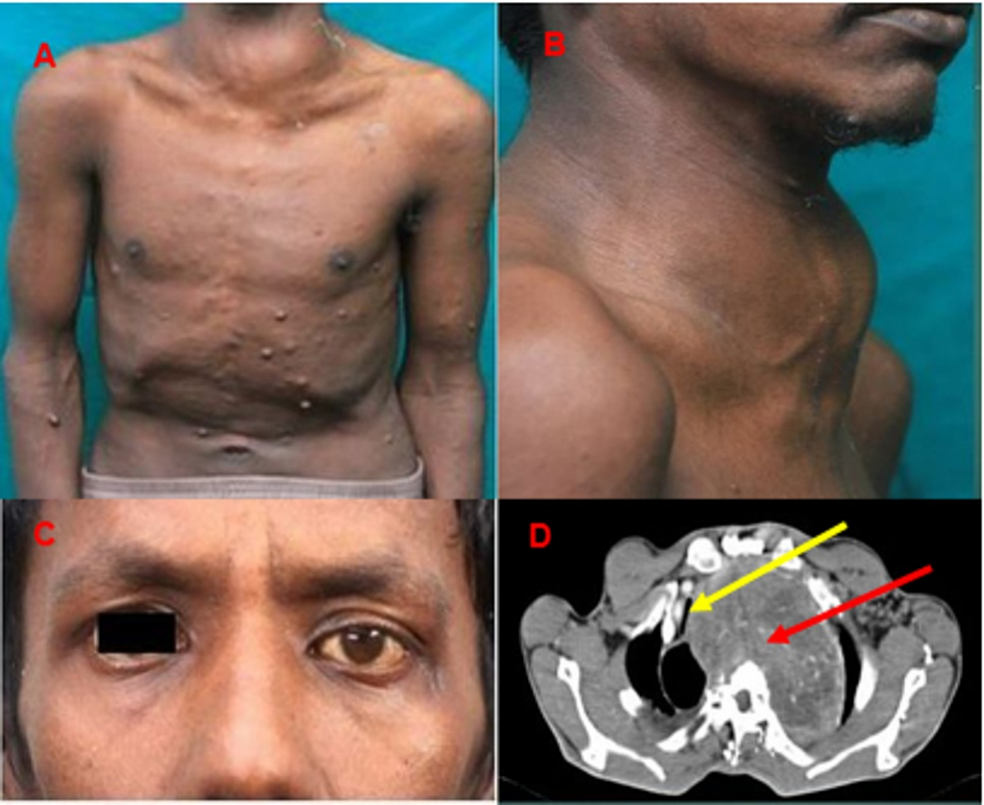
Clinical and radiological findings. The clinical photograph (A) shows multiple nodules over the chest wall, arms, and forearms consistent with neurofibromatosis. (B) shows a large swelling over the anterolateral aspect of the neck on the left side. (C) shows meiosis and partial ptosis of the left eye. CT scan shows a large lobulated mass (red arrow) compressing the trachea (yellow arrow) giving a slit-like appearance (D).

On examination, he had a large swelling (Figure 1B) over the anterolateral aspect of the left side of the neck, about 6 cm × 10 cm in size, which was non-tender on palpation. He had multiple neurofibromas on the skin (Figure 1A), multiple café-au-lait spots of size >2 cm in diameter, and lisch nodules on slit-lamp examination. Neurological examination revealed left eye enophthalmos, partial ptosis, and meiosis (Figure 1C). The right eye was normal. Extraocular movements in both eyes were normal. Other cranial nerves were normal. Motor system examination revealed clawing of the left little and ring fingers. There was a weakness of the small muscles of the hands. The card test was positive, suggesting weakness of palmar interossei. Dorsal interossei were also weak, Froment’s sign was positive in the left hand, suggesting weakness of adductor pollicis. Sensory system examination revealed loss of sensation in the left little, ring finger, hypothenar eminence, medial side of the arm, and forearm. The examination of other systems was normal. Overall, the clinical findings were consistent with Horner′s syndrome and left brachial plexopathy (lower trunk).

A contrast-enhanced CT scan of the neck, thorax, and abdomen revealed a large well-defined lobulated mass in the neck and superior mediastinum measuring 11 cm × 10 cm × 6 cm. The mass is abutting and compressing the esophagus, left main bronchus, and trachea (Figure 1D).

**Figure 2:**
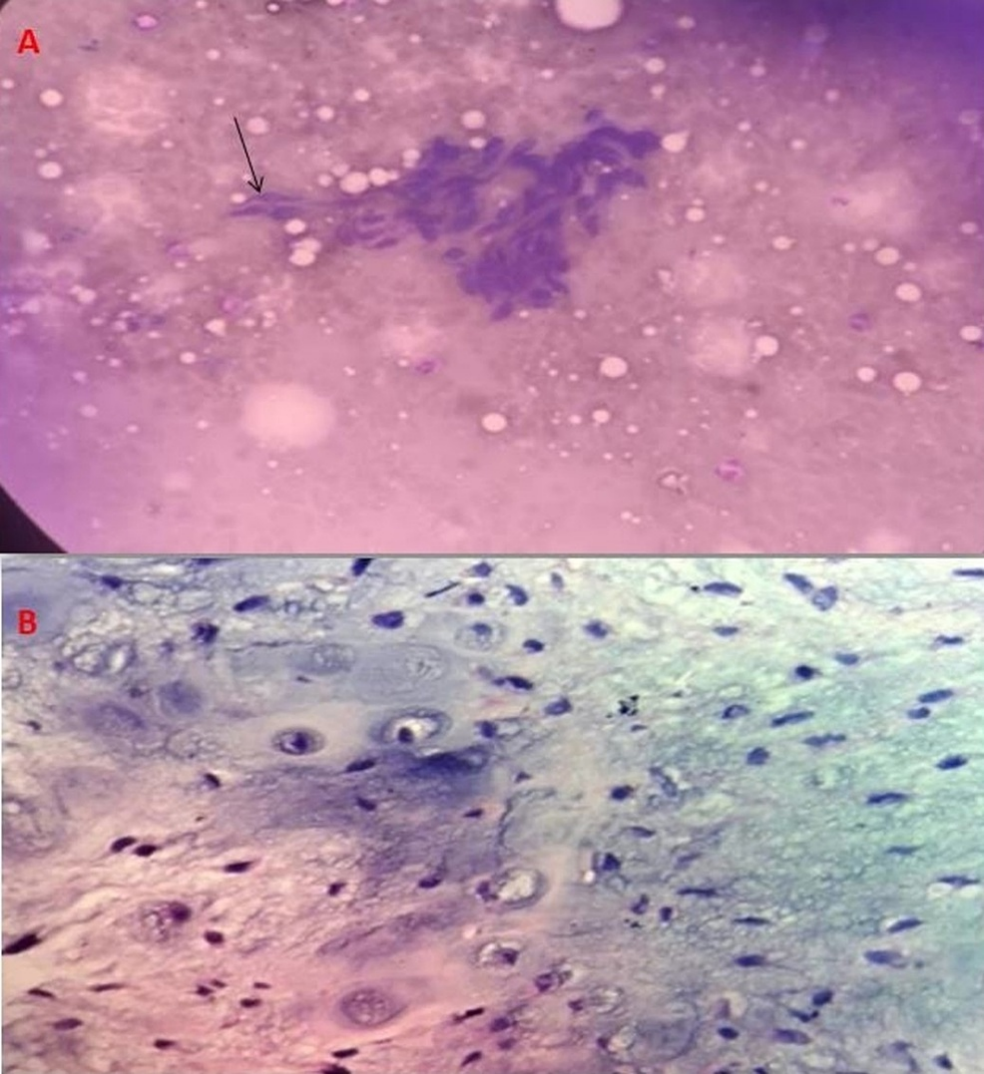
Histopathological examination. Fine needle aspiration cytology from the neck swelling (May Grunwald-Giemsa stain 40x magnification) showed spindle-shaped cells (black arrow) with fibromyxoid stroma (A). Histopathological examination of biopsy from the swelling (H&E stain) showed spindle cells with wavy nuclei, nuclear pleomorphism with infrequent mitotic figures, consistent with low-grade malignant peripheral nerve sheath tumor (B).

Fine needle aspiration cytology from the neck swelling (Figure 2A) showed spindle-shaped cells with fibromyxoid stromal matrix and many scattered tumor cells with moderate atypia. Biopsy from the mass (Figure 2B) was consistent with a low-grade MPNST. Immunohistochemistry of the tumor cells was negative for S 100. Ki-67 is 12%.

The patient underwent surgical excision of the mass. It was a stage 1 disease (American Joint Committee on Cancer Staging System for Soft Tissue Sarcoma). Post-surgery, he received adjuvant chemotherapy with doxorubicin and dacarbazine. Breathlessness and dysphagia have improved dramatically following surgery. However, features of Horner′s syndrome persisted. He has now completed eight months of follow-up.

## Discussion

NF1 is an autosomal dominant condition that affects one in 2500 to one in 3000 irrespective of sex or ethnicity [2]. NF1 patients are prone to benign and malignant tumors of the nervous system [3]. MPNST is a highly aggressive soft tissue sarcoma. The lifetime risk of developing MPNST in NF1 patients is 8%-13%. Sporadic cases usually occur between 30 and 60 years, however, NF1 associated MPNST presents between 20 and 40 years [4]. Horner′s syndrome is characterized by ptosis, meiosis, and anhydrosis. The lesion in Horner´s syndrome can be central, preganglionic, or postganglionic. The preganglionic sympathetic neurons originate in the ciliospinal center of Budge between the eighth cervical vertebra and the first thoracic vertebra of the spinal cord and synapse to the superior cervical ganglion. Lesion of the sympathetic chain at the preganglionic level often produces the full symptoms of Horner′s syndrome, including anhydrosis, although it can be seen in central Horner′s also. Preganglionic sympathetic lesions most commonly result from thoracic or cervical tumors [1, 5].

The presence of Horner′s syndrome in our case was due to the interruption of preganglionic occulosympathetic fibers by MPNST which is arising from the mediastinum. The apical bronchogenic carcinoma is the most common cause of Horner′s syndrome with brachial plexopathy [6]. MPNST in the case of NF1 is a very rare cause of Horner′s syndrome associated with brachial plexopathy [3], only one case has been reported so far [7]. Brachial plexopathy in our case is due to compression of the lower trunk by MPNST. To the best of our knowledge, this is the first case to be reported as a malignant peripheral nerve sheath tumor arising from mediastinum presenting as neck swelling and Horner′s syndrome. This case report expands the knowledge about the clinical behavior of NF1. This also shows us the importance of clinical examination and clinicians should be vigilant about the possibility of MPNST in NF1 patients presenting with features of Horner′s syndrome and neck swelling.

The genetic basis has been very well described for the association of NF1 with MPNST. Patients with NF1 start life with one mutant and one normal copy of the NF1 gene in the cells within their body. Preneoplastic Schwann cell precursors undergo a somatic NF1 loss, resulting in bi-allelic NF1 inactivation and benign neurofibroma formation [8]. Loss of CDKN2A leads to atypical neurofibroma formation, and mutations in other genes, including TP53, EGFR, and SUZ12, lead to MPNST formation [9-10].

Complete surgical resection with negative margins remains the only curative treatment to date [11], although it may not be possible always due to tumor location or size. In our case, the tumor was very large and abutting the great vessels, hence complete resection with negative margins could not be achieved in a few areas. The use of neoadjuvant chemotherapy is still under debate. Several studies have failed to show survival benefits after chemotherapy in MPNST [12-13]. However, most of the studies were small, retrospective, and often pooling data from multiple trials from multiple institutions. The most commonly used drugs in these trials were ifosfamide and doxorubicin. In all the previous studies majority of the cases were sporadic MPNST than NF1-associated MPNST. Hence the data regarding chemotherapy regimen for NF1 associated MPNST is lacking. All the previous studies have shown doxorubicin-based treatments have better outcomes in MPNST. The combination of doxorubicin and ifosfamide has a higher number of side effects than doxorubicin plus dacarbazine [14-15]. Hence, we used dacarbazine and there was not much of a difference in survival outcome. The role of adjuvant radiotherapy is not well-defined, may be used in cases with large tumor sizes. However, radiotherapy has not shown survival benefits [16]. The 10-year overall survival rate of NF1 associated MPNST is 34%-56% [17].

## Conclusions

Malignant peripheral nerve sheath tumor is a rare soft tissue sarcoma in the head and neck region. The presence of Horner′s syndrome and large neck swelling in NF1 patients should raise the suspicion of a MPNST. The prognosis of MPNST is poor. Although it was a late presentation, our patient improved well with surgery and chemotherapy. Our knowledge about genetic profiles has increased over the past decades; targeted molecular therapies following surgery are under development, rendering disease-free in near future.

## References

[1] Bilgic B, Ates LE, Demiryont M, Ozger H, Dizdar Y (2003). Malignant peripheral nerve sheath tumors associated with neurofibromatosis type 1. Pathol Oncol Res.

[2] Evans DG, Howard E, Giblin C (2010). Birth incidence and prevalence of tumor-prone syndromes: estimates from a UK family genetic register service. Am J Med Genet A.

[3] Williams VC, Lucas J, Babcock MA, Gutmann DH, Korf B, Maria BL (2009). Neurofibromatosis type 1 revisited. Pediatrics.

[4] Widemann BC (2009). Current status of sporadic and neurofibromatosis type 1-associated malignant peripheral nerve sheath tumors. Curr Oncol Rep.

[5] Cackett P, Vallance J, Bennett H (2005). Neurofibromatosis type 1 presenting with Horner's syndrome. Eye (Lond).

[6] Walker L, French S (2014). Horner's syndrome: a case report and review of the pathophysiology and clinical features. West Indian Med J.

[7] Basuthakur S, Sengupta A, Bandyopadhyay A, Banerjee A (2013). Malignant peripheral nerve sheath tumor presenting with Horner's syndrome. J Assoc Phys India.

[8] Zheng H, Chang L, Patel N, Yang J, Lowe L, Burns DK, Zhu Y (2008). Induction of abnormal proliferation by nonmyelinating Schwann cells triggers neurofibroma formation. Cancer Cell.

[9] Perry A, Kunz SN, Fuller CE (2002). Differential NF1, p16, and EGFR patterns by interphase cytogenetics (FISH) in malignant peripheral nerve sheath tumor (MPNST) and morphologically similar spindle cell neoplasms. J Neuropathol Exp Neurol.

[10] Zhang M, Wang Y, Jones S (2014). Somatic mutations of SUZ12 in malignant peripheral nerve sheath tumors. Nat Genet.

[11] Dunn GP, Spiliopoulos K, Plotkin SR, Hornicek FJ, Harmon DC, Delaney TF, Williams Z (2013). Role of resection of malignant peripheral nerve sheath tumors in patients with neurofibromatosis type 1. J Neurosurg.

[12] Ferrari A, Miceli R, Rey A (2011). Non-metastatic unresected paediatric non-rhabdomyosarcoma soft tissue sarcomas: results of a pooled analysis from United States and European groups. Eur J Cancer.

[13] Kroep JR, Ouali M, Gelderblom H (2011). First-line chemotherapy for malignant peripheral nerve sheath tumor (MPNST) versus other histological soft tissue sarcoma subtypes and as a prognostic factor for MPNST: an EORTC soft tissue and bone sarcoma group study. Ann Oncol.

[14] Edmonson JH, Ryan LM, Blum RH (1993). Randomized comparison of doxorubicin alone versus ifosfamide plus doxorubicin or mitomycin, doxorubicin, and cisplatin against advanced soft tissue sarcomas. J Clin Oncol.

[15] Judson I, Verweij J, Gelderblom H (2014). European Organisation and Treatment of Cancer Soft Tissue and Bone Sarcoma Group. Doxorubicin alone versus intensified doxorubicin plus ifosfamide for first-line treatment of advanced or metastatic soft-tissue sarcoma: a randomized controlled phase 3 trial. Lancet Oncol.

[16] Kahn J, Gillespie A, Tsokos M (2014). Radiation therapy in management of sporadic and neurofibromatosis type 1-associated malignant peripheral nerve sheath tumors. Front Oncol.

[17] Hwang IK, Hahn SM, Kim HS (2017). Outcomes of treatment for malignant peripheral nerve sheath tumors: different clinical features associated with neurofibromatosis type 1. Cancer Res Treat.

